# Bias-dependent local structure of water molecules at a metallic interface†
†Electronic supplementary information (ESI) available. See DOI: 10.1039/c7sc02208e


**DOI:** 10.1039/c7sc02208e

**Published:** 2017-10-11

**Authors:** Luana S. Pedroza, Pedro Brandimarte, Alexandre Reily Rocha, M.-V. Fernández-Serra

**Affiliations:** a ICTP South American Institute for Fundamental Research , Instituto de Física Teórica , Universidade Estadual Paulista , São Paulo SP 01140-070 , Brazil . Email: l.pedroza@ufabc.edu.br; b Centro de Ciências Naturais e Humanas , Universidade Federal do ABC , Santo André , São Paulo , Brazil 09210-170; c Centro de Física de Materiales , 20018 Donostia – San Sebastián , Gipuzkoa , Spain; d Donostia International Physics Center , 20018 Donostia – San Sebastián , Gipuzkoa , Spain; e Instituto de Física Teórica , Universidade Estadual Paulista , São Paulo SP 01140-070 , Brazil; f Department of Physics and Astronomy , Stony Brook University , Stony Brook , New York 11794-3800 , USA; g Institute for Advanced Computational Sciences , Stony Brook University , Stony Brook , New York 11794-3800 , USA

## Abstract

Understanding the local structure of water at the interfaces of metallic electrodes is a key issue in aqueous-based electrochemistry. Nevertheless a realistic simulation of such a setup is challenging, particularly when the electrodes are maintained at different potentials. To correctly compute the effect of an external bias potential applied to truly semi-infinite surfaces, we combine Density Functional Theory (DFT) and Non-Equilibrium Green’s Function (NEGF) methods. This framework allows for the out-of-equilibrium calculation of forces and dynamics, and directly correlates to the chemical potential of the electrodes, which is introduced experimentally. In this work, we apply this methodology to study the electronic properties and atomic forces of a water molecule at the interface of a gold surface. We find that the water molecule tends to align its dipole moment with the electric field, and it is either repelled or attracted to the metal depending on the sign and magnitude of the applied bias, in an asymmetric fashion.

## Abstract 2

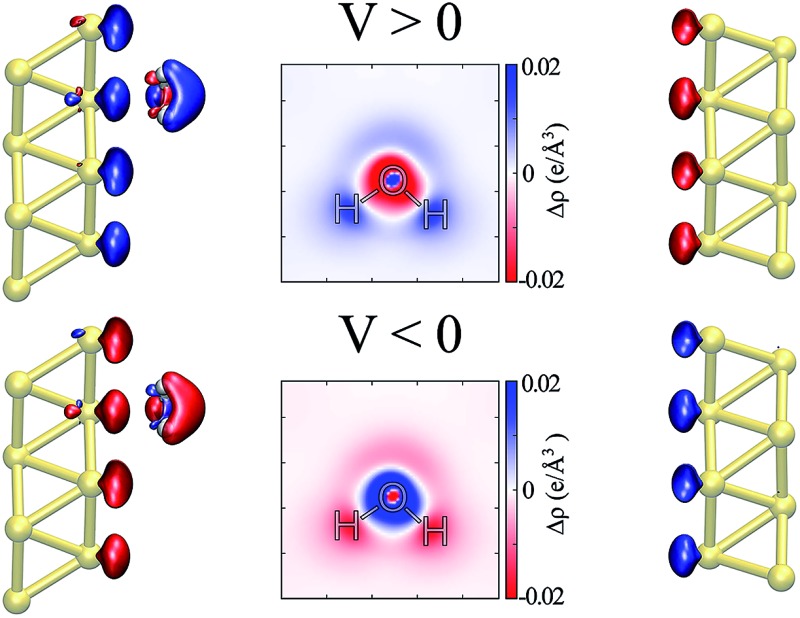
We combine Density Functional Theory (DFT) and Non-Equilibrium Green’s Function (NEGF) methods to study the electronic properties and atomic forces of a water molecule at metallic interfaces.

## 1 Introduction

Following the need for new and renewable sources of energy worldwide, fuel cells using electrocatalysts can be thought of as viable options.^1^,^2^ The interface between metal (electrode) and water in these systems is the electrochemical central point, since it is the region where charge transfer can take place. A better understanding of the metal–water interface is also an essential requisite for predicting the correspondence between the macroscopic voltage and the microscopic interfacial charge distribution in electrochemical fuel cells. This reactivity is governed by the explicit atomic and electronic structures built at the interface as a response to external conditions, such as an applied potential.^3^–^5^

The advance in experimental techniques for studying surfaces in the last decades started to provide important results concerning the local structure of water at interfaces, revealing a bias-dependent behavior.^6^–^8^ Notably, in a more realistic system applied to catalysis, the metal will be at a given potential. From a theoretical perspective this makes the task of simulating this setup difficult.^9^ In fact, accurate calculation of the electrostatic potential at electrically-biased metal–electrolyte interfaces is a challenge for *ab initio* simulations with periodic boundary conditions.^10^,^11^

One possible way to simulate this electrochemical cell under an explicit bias is to account for the polarization of the metal by charging each atom on the electrode, enforcing a constant potential and using the image charges method.^12^–^14^ Although this can provide interesting insights into the problem, the characterization is limited to the use of empirical models. Neurock’s studies of water/metal interfaces in the presence of an applied potential^15^,^16^ were among the first ones to use Density Functional Theory (DFT)^17^,^18^ to address this problem. The resulting electrode potential – which is related to the Fermi energy of the system – is compared to an internal reference potential by artificially inserting a vacuum layer into the center of the solution region. To be able to fully compute all of these energies when the system is charged, the water molecules in the center of the liquid layer are usually held fixed during the optimization of the charged systems. More recently, N. Bonnet *et al.* proposed a methodology where an external potentiostat was added to the system^19^ following a previous work where the charges at the surface were controlled by including a medium with a given permittivity.^20^ The idea in the later work was to use the potential energy of a fictitious system, akin to the fictitious mass in Car–Parrinello first-principles molecular dynamics. Therefore, current methodologies^21^ attempt to simulate the effect of finite bias at the metal by altering their charge (adding/subtracting electrons), whereas in experiments the potential is the quantity that is controlled.

The electrochemical cell can be thought of as two metallic electrodes which act as charge reservoirs, with the two metal plates separated by a solution (mostly composed of water). This arrangement is analogous to the one encountered in simulations of electronic transport: a central scattering region coupled to electrodes.^22^–^24^ Thus, we propose in this work, as an alternative, to use open boundary conditions by employing the non-equilibrium Green’s function (NEGF) method combined with DFT to properly compute the effect of an external bias potential applied to electrodes. While standard DFT implementations are not suited to treating extended systems under an external bias, NEGF has been designed to treat out-of-equilibrium situations. Most notably it allows for the inclusion of truly semi-infinite metallic electrodes, which set the correct chemical potential for the metal, and a clear reference potential. Their combination has been developed over the past decade to describe the current–voltage characteristics of nanoscopic systems.^23^–^25^ It treats an open system under the influence of an external bias, and although dynamics – or forces – are typically ignored in such systems, they can be incorporated into the methodology. In this work, we apply this framework to a system consisting of a single water molecule between two Au(111) surfaces, at different configurations and as a function of an external voltage.

## 2 Methodology

### 2.1 General methodology

**Fig. 1:**
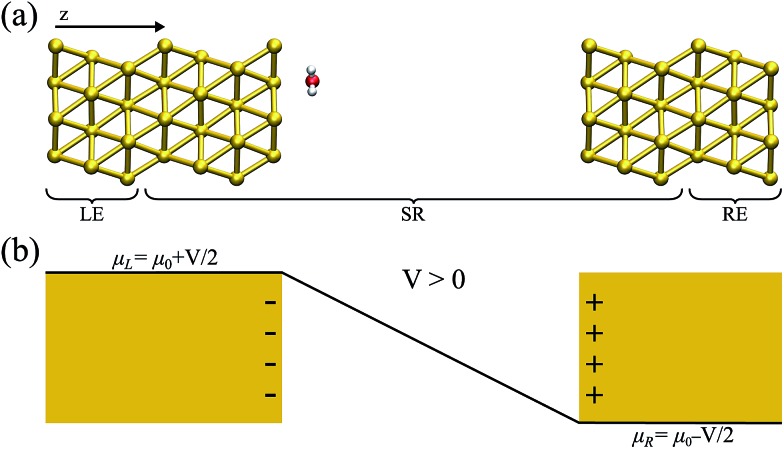
(a) A schematic view of the metal–water system used for the non-equilibrium calculations; the left (right) electrodes (LE/RE) and scattering region (SR) are indicated. (b) A sketch of the effect of a positive bias potential on a parallel plate capacitor; the corresponding charge accumulated in each plate, as well as the bias ramp, is shown.

In what has become the usual approach in electronic transport calculations, the system is divided into three regions: electrodes (left and right, L/R) and scattering region (SR).^26^ We framed our metal–water system into an analogous arrangement. In this case, the interfacial region (metal–water–metal) represented the scattering region. The first few layers of the metal at the interface needed to be considered part of the scattering region as we required that the charge density at the edge of the SR resembled that of the bulk metal. Then, the electronic charge distributions in the electrodes, left and right, corresponded to the bulk phases of the same material to a prescribed numerical accuracy. A representation of the typical arrangement used in our calculations is shown in Fig. 1(a).

When a finite voltage is applied to the electrodes the problem becomes a non-equilibrium one. The electrodes are then ascribed different chemical potentials and current, in principle, can flow. The non-equilibrium Green’s function formalism is a general formalism for calculating the properties of systems in out-of-equilibrium situations, and can be used to tackle our problem of the electrochemical cell. In principle it can be used to address problems where inelastic effects are present, and most importantly, it goes far beyond electronic transport (including ballistic transport).

Within the NEGF approach, if the Hamiltonian can be cast in a bilinear form, the entire problem can be treated in terms of a single Green’s function; in our case the retarded Green’s function for the scattering region is:1
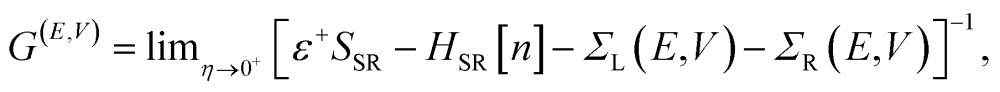
where *ε*^+^ = *E* + *iη*, *S*_SR_ is the overlap matrix and *H*_SR_[*n*] is a Hamiltonian which is a functional of the charge density, *n*(*r*). In this work the Hamiltonian was taken as the Kohn–Sham (KS) Hamiltonian from DFT. The effects of the electrodes were introduced in the form of the self-energies *Σ*_L/R_, which were obtained by integrating out the degrees of freedom of the leads. As the electrodes were considered to be good metals, the effect of the bias on the left and right electrodes corresponds to a rigid shift (±*V*/2) of the zero-bias self-energies, setting the boundary condition (illustrated in Fig. 1(b) for a positive bias). This means that the self-energies could be obtained from a separate DFT calculation for the bulk metal, and did not need to be updated self-consistently throughout the calculation. This approach also ensured that a clear reference potential was defined (the chemical *μ*_0_ of the bulk metal) as we assumed the electrodes were charge reservoirs in thermodynamic equilibrium throughout the calculations.

Once the Green’s function of the SR was calculated, all of the observables of the system could be recomputed. In particular the density matrix is expressed as:2
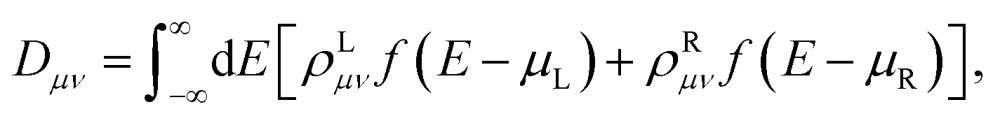
where the *μ* and *ν* indices run over the SR electronic states, *f*(*E*) = 1/(1 + e^*E*/*kT*^) is the Fermi distribution and *μ*_R_ and *μ*_L_ are the electrochemical potentials of the right and left electrodes (*μ*_L/R_ = *μ*_0_ ± *V*/2) that define the bias: *V* = *μ*_L_ – *μ*_R_. Finally,

3 *ρ*^L/R^ = *G*(*E*)*Γ*_L/R_(*E*)*G*^†^(*E*)

is the electrode spectral density matrix, obtained from the Green’s function of the SR and the left (right) coupling matrices, 
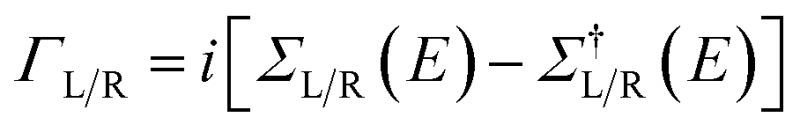
. From the density matrix, the KS Hamiltonian could be computed. The procedure was then repeated until self-consistency was achieved.

Within the ground state DFT framework, the computation of forces on the nuclei was theoretically well-founded thanks to the Hellmann–Feynman theorem,^27^,^28^ and the forces were obtained *via* the derivative of the total energy. Using a set of localized basis functions the force was decomposed into two terms:^29^ one that originated from the derivative of the energy of the occupied eigenstates (band structure contribution) and a second one that contained the remaining contributions to the energy. For an ion I, the former one was given by:4
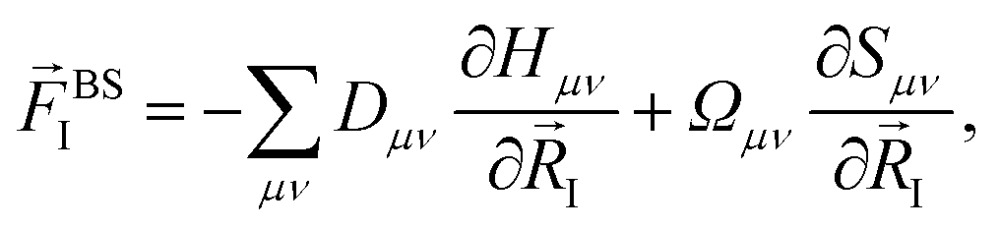
where *D*_*μν*_ is the density matrix and *Ω*_*μν*_ is the energy density matrix 
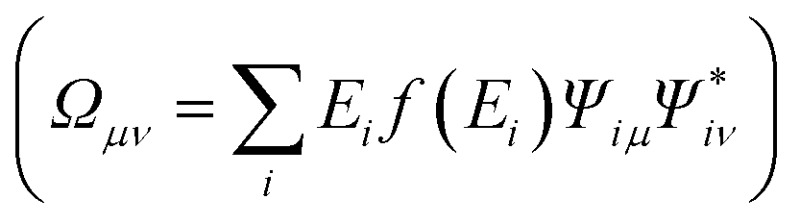
. The situation is more complex out of equilibrium, where the Hellmann–Feynman theorem does not apply.^30^ Recently, it was shown that the forces can actually be obtained by the time derivative of the expectation value of the ionic momentum operators:^29^,^30^
5
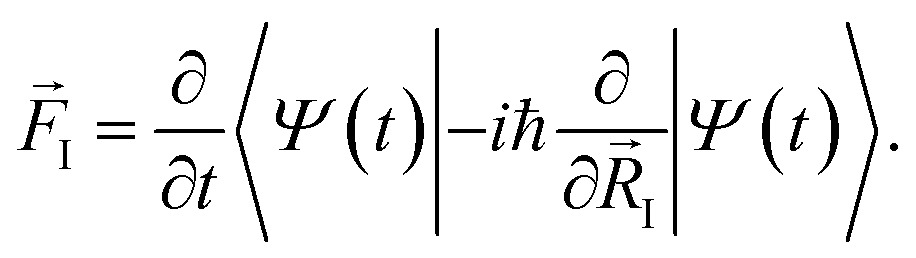



As it turns out, for steady-state problems, the final form for the force is equivalent to the equilibrium case:6
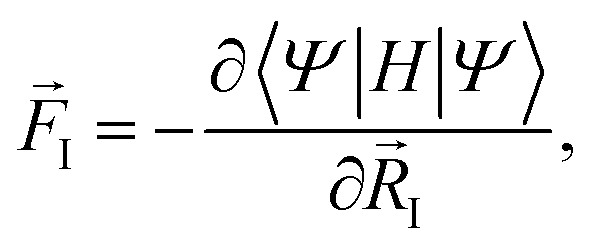
which can be expressed by eqn (4), replacing the ground state density matrix and energy density matrix with the out-of-equilibrium ones, obtained in terms of the retarded Green’s function.

One important point that still remains is how well defined the forces are when current flows in the system.^31^–^33^ In our case, however, the gap in the scattering region – the band gap of water – was large enough (∼8 eV) to ensure that no current would flow through the arrangement. In that sense, it is important to stress that current-induced forces were not present in our problem. This remains true for the simulation of ionic electrolytes, where ionic currents are expected to exist – and can be captured by this method – but no electronic currents exist.

Finally, one notices that the above methodology relies on the Kohn–Sham Hamiltonian being a good description of the single particle excitations for the system, as is the case in different implementations of the NEGF formalism within DFT.^23^,^24^ This leads to known pitfalls, which are associated with the positions of the molecular energy levels and charge transfer between the surfaces and molecules to mention a few.^34^,^35^ Most of these issues however, pertain to approximations in the exchange–correlation functional, and corrections in different forms can be readily incorporated into the formalism.^36^–^40^ Nonetheless, it is important to point out that local and semi-local functionals tend to perform better for forces and structures compared to total energies and single particle energy levels.

The described methodology was implemented in the Smeagol code^24^,^25^ which was bundled with Siesta.^41^,^42^ In the same way that relaxation and *ab initio* molecular dynamics can be performed within DFT, one can now use the code to do the same for out-of-equilibrium systems.

### 2.2 Details of calculations

In this work, we used two different gradient-dependent exchange–correlation (XC) functionals: PBE^43^ and vdW-DF^PBE^, which includes van der Waals corrections (vdW). The vdW-DF^PBE^ functional is a modified version of the original vdW-DF functional,^44^ in which the revPBE local term is replaced by PBE.^45^ The core electrons were described by norm-conserving pseudopotentials in the Troullier–Martins form.^46^ A basis set of numerical atomic orbitals with double-*ζ* polarization was used to describe the valence electrons. For both the metal and water the basis set was variationally optimized and ensured that our results (Au lattice parameter and water–metal geometry) were in agreement with the plane-wave calculations.

For the non-equilibrium calculations, each metal slab within the scattering region had 3 layers of (111) planes with 12 Au atoms on each plane of a size of 10.29 × 9.89 Å in the plane perpendicular to the transport direction. This size was chosen because periodic boundary conditions are still applied in this plane, and it is necessary to minimize interaction between periodic repetitions of the water molecules in the plane. The water molecule was placed close to one metal surface (the one defined as the left). In order to minimize the interaction between the surfaces, the right and left side were 20 Å apart. The electrodes, connected to the scattering region, consisted of 3 Au layers each (left and right). Fig. 1 shows a schematic view of the system and its components.

## 3 Results

**Fig. 2:**
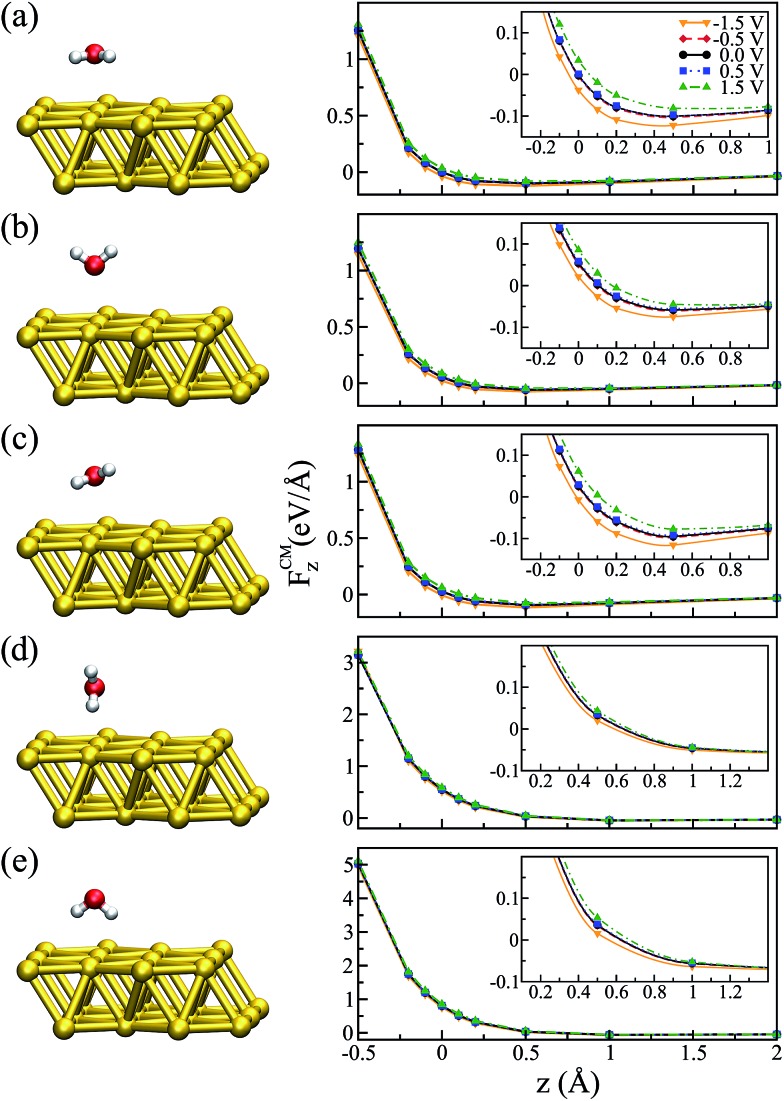
*z*-Component of the force at the center of mass (right panels) as a function of the vertical displacement for different water configurations (left panels): (a) “flat”, (b) “up”, (c) “flat-up”, (d) “perpendicular” and (e) “down”. The vertical displacement is given with respect to the Au–O distance in the ground state. The insets of the right panels show the regions in each graph for which *F*CM*z* = 0.

Before applying a bias at the electrodes it was important to characterize the ground state configuration of the metal–water system. This was initially done using a (111) surface Au slab with 4 layers and a 2 × 2 in-plane supercell within the standard periodic DFT formalism. The relaxed water structures were then used as starting configurations for the larger gold surfaces. All of the atoms were allowed to move during the geometrical optimization using the conjugate gradient algorithm and with a 0.005 eV Å^–1^ tolerance criteria on the forces. The final configurations were very similar to the ones used as a starting point. Our results show that the most stable configuration of the water molecule on top of the Au(111) surface is the so-called “flat” one (*i.e.* the molecule dipole moment is almost parallel to the surface plane), in agreement with previously reported calculations.^47^,^48^ The molecular plane was slightly tilted with respect to the metal plane, with *α* = 3° (*α* being the angle between the molecular plane and the surface plane) and the distance between Au and O *d*_Au–O_ = 2.92 Å. This structure is illustrated in Fig. 2(a). We also relaxed the metal–water–metal structure using the NEGF formalism at zero bias, obtaining *α* = 6° and 2.79 Å for the Au–O distance. The small differences are attributed to the effect of using a finite representation of Au surface in the standard DFT calculation.

It is worth mentioning that the potential energy surface (PES) for this system was very flat in the region of the water on top of the Au. For instance, the energy difference between the configuration where the molecule was “flat” and the one where it had the hydrogens pointing down (towards the metal) was only ∼0.06 eV. Therefore, we also considered four other different configurations for the water molecule, corresponding to rigid rotations of the ground state structure (left panels of Fig. 2). The geometries were labeled according to their orientation: “up” (hydrogens pointing away from the metal), “down” (hydrogens pointing towards the metal), “perpendicular” (*α* = 90° and *θ* = 90°), and “flat-up” (one hydrogen higher than the flat configuration, with *α* = 24° and *θ* = 84°). The angle *θ* corresponded to the angle between the molecule dipole and surface normal.

In order to analyze the effect of different bias voltages (magnitude and sign) on the molecule as a function of the distance of the water molecules to the metal, we first performed non-equilibrium calculations for all of the water structures. For each configuration, we started at the ground state Au–O distance (*z* = 2.79 Å, which corresponds to zero in the plots) at zero bias and increased/decreased this distance by –0.5 to +2.0 Å, and performed a single point calculation. At each point the forces on the atoms were evaluated for a particular applied bias. Since the water molecule was placed close to one metal surface, we observed that the potential in the water molecule closely followed that of the surface. Therefore, the bias indicated in the plots corresponds to *V*/2, as it corresponds to the potential that effectively acted on the molecule.

The results for the *z*-component of the force on the center of mass of the molecule, *F*CM*z*, are shown in Fig. 2 for all orientations. In general, we observed that low bias (–0.5 and +0.5 V) had a small effect on the forces, independent of the water configuration. However, as we increased the applied bias we observed that the forces close to the minimum were modified. In particular, this effect was more evident for the flat molecule (Fig. 2(a)) and for the configurations where the oxygen was facing the metal, due to strong interaction between the oxygen-b_1_ orbital of the water molecule and the metal orbitals.^49^,^50^

Fig. 2 indicates that there was a tendency to modify the position of the minimum configuration when the bias was applied. Moreover, this modification was dependent on the magnitude of the bias and it was asymmetric with respect to positive and negative values. This behavior is similar to what was verified when an electric field was applied:^51^,^52^ the molecule tended to get closer to the metal when the bias was negative and moved away from the metal with a positive bias, evidenced by the position at which *F*CM*z* = 0. A similar trend can be observed for the “up” and “flat-up” configurations shown in Fig. 2(b and c), respectively. For the “perpendicular” and “down” configurations the molecule was essentially unbound (Fig. 2(d and e), respectively).

**Fig. 3:**
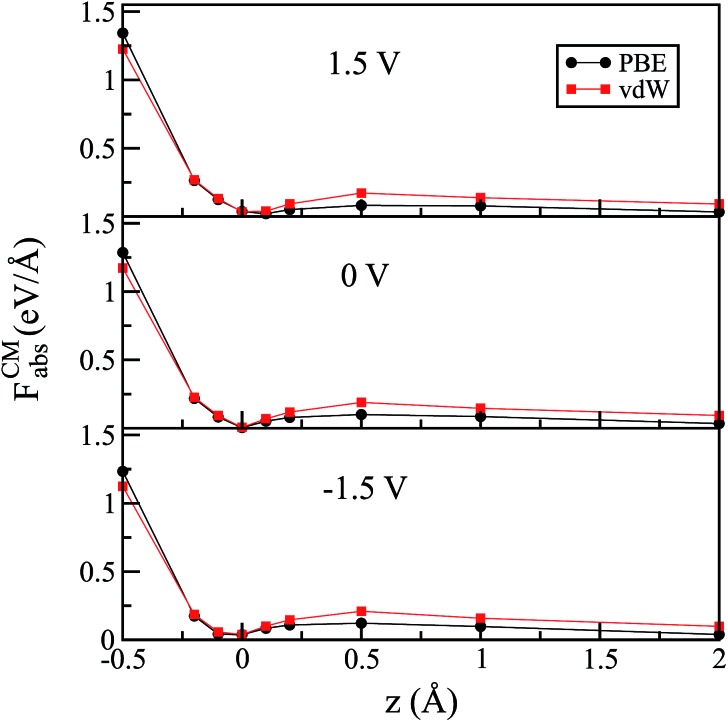
The magnitude of the force at the center of mass as a function of the vertical displacement for the “flat” configuration for the PBE and vdW-DF^PBE^ exchange–correlation functionals. The vertical displacement is given with respect to the Au–O distance at ground states corresponding to each functional.

A similar behavior was also observed when vdW corrections were included for the flat configuration, as shown in Fig. 3. In agreement with previous work,^53^ we note that the vdW functional did not significantly change the water–Au interaction. We observe, however, that the barrier in all of the cases increased slightly for higher distances; this is true for zero as well as for finite (positive or negative) bias. This means that, although the equilibrium position of the molecule did not depend on the choice of XC functional (specifically for Au–water systems), the restoration force was larger when we included vdW interactions.

**Fig. 4:**
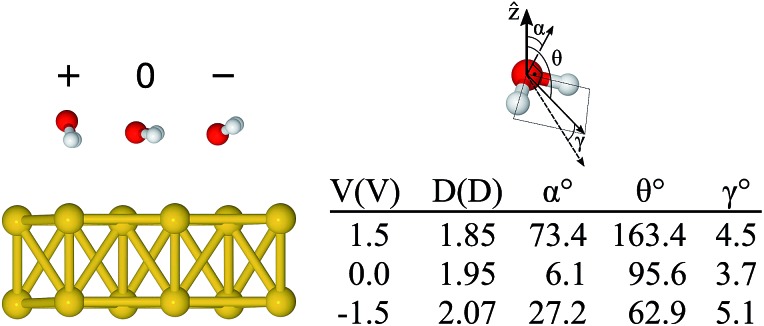
Relaxed configurations of the water molecule on the Au surface for different bias voltages (+1.5 V → +, 0 V, and –1.5 V → –), the corresponding angles, and the estimated dipole moment (details in ESI†). The angle *α* is the angle between the molecular plane and the surface plane, *θ* is the angle between the isolated molecule dipole and the surface normal, and *γ* is the angle between the isolated molecule dipole and that calculated from the charge density of the combined Au + H_2_O system.

Although by rigidly shifting the position of the molecule along *z*, one can always find a position for which *F*CM*z* = 0, that is not the case for all directions concomitantly (see ESI†). This is an indication that, as the absolute value of the bias increases, the orientation of the molecule tends to change as well. Thus, in a second step, starting from the “flat” configuration, we allowed the atoms of the water molecule to move using the conjugate gradient algorithm with a bias applied to the system. The minimum configurations obtained for –1.5 and +1.5 V are shown in Fig. 4, where the geometry obtained for the zero bias case is also shown. The asymmetric behaviour with respect to the bias sign was clearly observed. The geometry for +1.5 V had the hydrogen atoms pointing down and the oxygen atom was 2.84 Å away from the metal. In fact, this is the “down” configuration (*α* = 90° and *θ* = 180°), presented in Fig. 2(e), which means that the positive bias leads to an unbound molecule. On the other hand, when the negative bias was applied the hydrogen atoms moved slightly upwards (*α* = 27° and *θ* = 63°) and the oxygen got closer to the metal (*d*_O–Au_ = 2.69 Å) compared to the neutral case. In essence, one notices that even a relatively small bias can lead to significant structural changes on the metal–water interface.

**Fig. 5:**
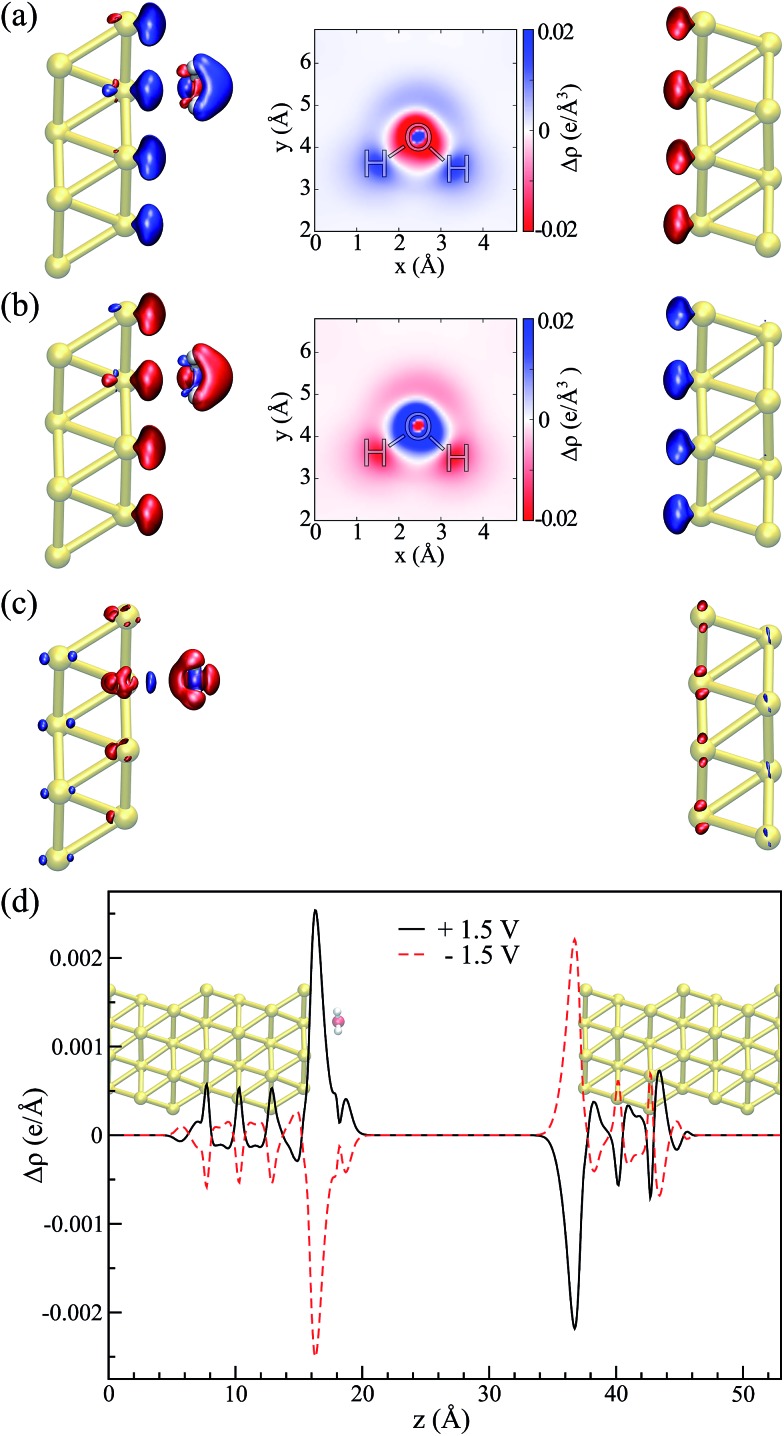
Flat configuration. Relative changes in the charge density in comparison to the zero bias, Δ*ρ*_*V*,0_ = *ρ*_*V*_ – *ρ*_0_ for (a) *V* = +1.5 V and (b) *V* = –1.5 V (isosurface values = ±8.1 × 10^–4^ e per Å^3^). Insets show an *xy*-sectional plane taken at the water molecule center of mass. (c) Charge density fluctuation at positive and negative applied biases, *i.e.* ΣΔ*ρ* = Δ*ρ*_1.5,0_ + Δ*ρ*_–1.5,0_ (isosurface value = ±5.4 × 10^–5^ e per Å^3^). In all of the cases, red (blue) indicates an excess (deficiency) of electrons. (d) Laterally averaged difference in the charge density in comparison to the zero bias: black for +1.5 V and red for –1.5 V; the water–metal system indicates the position of the atoms.

The water–Au interaction is mostly electrostatic in nature, and there is almost no charge transfer between water and the metal in the neutral case,^53^ as seen from a Bader analysis^54^ of the cases with and without bias (see Fig. S2 of the ESI†). At the same time, the effect of the bias on configurations can be understood in terms of a combination of increase/decrease in Pauli repulsion and small charging of the surface. Fig. 5(a and b) show the differences in charge density for different biases compared to the zero-bias case for the “flat” configuration. The corresponding insets of Fig. 5 indicate that most of the change in charge on the molecule is located on the oxygen. That transfer is small, however, as seen in both the insets and Fig. 5(d) which shows the change in charge density averaged over planes perpendicular to the surface. At the same time, by calculating the fluctuations in the density differences between the positive and negative bias,

7 ΣΔ*ρ* = Δ*ρ*_1.5,0_ + Δ*ρ*_–1.5,0_

8 = (*ρ*_*V*=1.5_ – *ρ*_*V*=0_) + (*ρ*_*V*=–1.5_ – *ρ*_*V*=0_),

presented in Fig. 5(c), we notice that, although small (the value of the isosurface is 5.4 × 10^–5^ e per Å^3^), it is asymmetric. Furthermore the final values of the water molecule dipole moments are similar to those of the isolated molecule in the cases with and without bias, and those dipoles tend to align with the field. The estimated magnitude of the dipole moments as a function of the applied bias are presented in the table of Fig. 4 together with the angular deviation from the dipole of an isolated molecule with the same orientation.

**Fig. 6:**
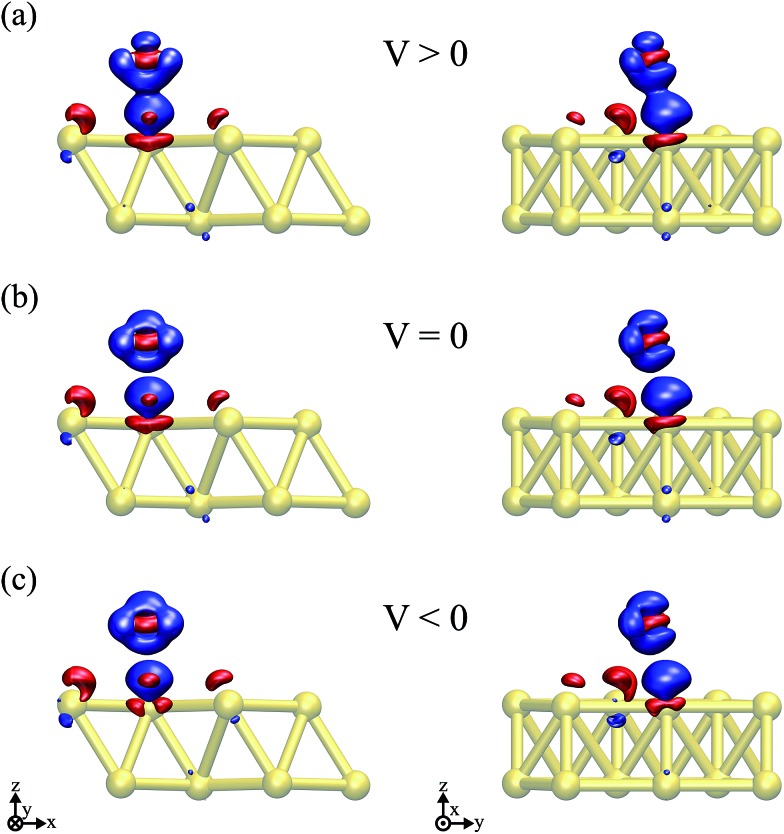
Differences in the charge density between the Au + water system, a parallel plate capacitor and an isolated H_2_O molecule submitted to an equivalent electric field for (a) V = 1.5 V, (b) V = 0, and (c) V = –1.5 V. An isosurface value of ±8.4 × 10^–3^ e per Å^3^ was considered in all of the plots, where red (blue) indicates an excess (deficiency) of electrons.

Thus, the picture that arises is the following: for the positive bias the left hand side surface becomes negatively charged and tends to repel the negatively charged oxygen. At the same time as a small amount of charge is transferred to the oxygen, the overlap between the oxygen orbitals and gold surface orbitals tends to move the molecule away due to Pauli repulsion. The opposite behavior is expected for the negative bias. This last point is evidenced in Fig. 6 where we show the difference between our Au–water system and the charge density in an isolated capacitor with a ±1.5 V bias applied and an isolated water molecule in an equivalent electric field. In doing this we remove effects from the charge density that would arise solely from the electric field, focusing instead on the effects due to the water–surface interaction, namely the Pauli repulsion. For zero bias (Fig. 6(b)) the signature of Pauli repulsion, namely the “pillow” density of states between the molecule and metallic surface, is already visible.^55^,^56^ For the positive bias, the interaction between molecule and surface increases and the nodes in the density disappear, an indication of decreased Pauli repulsion. The interaction is thus more attractive. On the other hand, for the negative bias there is larger repulsion as indicated by a slightly larger gap between the “pillow” region and the charge density associated with the molecule.

## 4 Conclusions

In conclusion, the inclusion of electronic effects *via* DFT in the description of water–metal interactions is important to advance the comprehension of the local structure of water at an electrochemical interface. In this work we showed how DFT combined with NEGF can be used to describe water–metal systems under an external bias potential. This framework allows for a description of a truly semi-infinite metallic electrode that sets a reference chemical potential that can be controlled by applying an external bias without adding/removing additional charge to the system. This allows for a more direct comparison with the experimental setups. This methodology now allows one to properly calculate the forces and therefore perform relaxation or dynamics of water–metal systems out of equilibrium, simulating an electrochemical cell in the sense that ionic currents could be considered now. This can be achieved by performing a molecular dynamics simulation of the system with the bias applied. In principle, our model allows one to consider a more realistic electrochemical cell with a thicker region of water molecules, although it will be computationally more expensive. One possible way to increase the system size in a cheaper way is to consider only the double layer region at the DFT level and the other water molecules at a molecular mechanics level (QM/MM method).^57^,^58^

Here, we presented how the magnitude and sign of the bias alters the interaction of a prototype system of one water molecule on top of an Au(111) surface. The external bias changes both the position and alignment of the molecule with the surface. In particular, we have showed that a small positive bias leads to an unbound water molecule on a gold surface due to a combination of electrostatic effects and Pauli repulsion. On the other hand, a negative bias increases the oxygen–metal bond, and leads to a slightly rotated water molecule, thus indicating that the introduction of an external bias has a significant influence on the microscopic structure of molecules at a metallic interface.

## Conflicts of interest

There are no conflicts to declare.

## Supplementary Material

Supplementary information

## References

[cit1] Norskov J. K., Bligaard T., Rossmeisl J., Christensen C. H. (2009). Nat. Chem..

[cit2] Greeley J., Markovic N. M. (2012). Energy Environ. Sci..

[cit3] Hodgson A., Haq S. (2009). Surf. Sci. Rep..

[cit4] Thiel P. A., Madley T. E. (1987). Surf. Sci. Rep..

[cit5] Björneholm O., Hansen M. H., Hodgson A., Liu L.-M., Limmer D. T., Michaelides A., Pedevilla P., Rossmeisl J., Shen H., Tocci G., Tyrode E., Walz M.-M., Werner J., Bluhm H. (2016). Chem. Rev..

[cit6] Toney M. F., Howard J. N., Richer J., Borges G. L., Gordon J. G., Meiroy O. R., Wiesler D. G., Yee D., Sorensen L. B. (1994). Nature.

[cit7] Velasco-Velez J.-J., Wu C. H., Pascal T. A., Wan L. F., Guo J., Prendergast D., Salmeron M. (2014). Science.

[cit8] Ataka K., Yotsuyanagi T., Osawa M. (1996). J. Phys. Chem..

[cit9] Nielsen M., Björketun M. E., Hansen M. H., Rossmeisl J. (2015). Surf. Sci..

[cit10] Calle-Vallejo F., Koper M. T. M. (2012). Electrochim. Acta.

[cit11] Cheng J., Sprik M. (2012). Phys. Chem. Chem. Phys..

[cit12] Siepmann J. I., Sprik M. (1995). J. Chem. Phys..

[cit13] Willard A. P., Reed S. K., Madden P. A., Chandler D. (2009). Faraday Discuss..

[cit14] Petersen M. K., Kumar R., White H. S., Voth G. A. (2012). J. Phys. Chem. C.

[cit15] Taylor C. D., Wasileski S. A., Filhol J.-S., Neurock M. (2006). Phys. Rev. B: Condens. Matter Mater. Phys..

[cit16] Filhol J.-S., Neurock M. (2006). Angew. Chem., Int. Ed..

[cit17] Hohenberg P., Kohn W. (1964). Phys. Rev..

[cit18] Kohn W., Sham L. J. (1965). Phys. Rev. A.

[cit19] Bonnet N., Morishita T., Sugino O., Otani M. (2012). Phys. Rev. Lett..

[cit20] Otani M., Sugino O. (2006). Phys. Rev. B: Condens. Matter Mater. Phys..

[cit21] Mamattkulov M., Filhol J.-S. (2011). Phys. Chem. Chem. Phys..

[cit22] Datta S., Electronic Transport in Mesoscopic Systems, Cambridge University Press, 1997.

[cit23] Brandbyge M., Mozos J.-L., Ordejón P., Taylor J., Stokbro K. (2002). Phys. Rev. B: Condens. Matter Mater. Phys..

[cit24] Rocha A. R., García-Suárez V. M., Bailey S., Lambert C., Ferrer J., Sanvito S. (2006). Phys. Rev. B: Condens. Matter Mater. Phys..

[cit25] Rocha A. R., García-Suárez V. M., Bailey S. W., Lambert C. J., Ferrer J., Sanvito S. (2005). Nat. Mater..

[cit26] Caroli C., Combescot R., Nozieres P., Saint-James D. (1971). J. Phys. C: Solid State Phys..

[cit27] Hellmann H., Einführung in die Quantenchemie, F. Deuticke, Leipzig, 1937, vol. 54.

[cit28] Feynman R. P. (1939). Phys. Rev..

[cit29] Zhang R., Rungger I., Sanvito S., Hou S. (2011). Phys. Rev. B: Condens. Matter Mater. Phys..

[cit30] Di Ventra M., Pantelides S. T. (2000). Phys. Rev. B: Condens. Matter Mater. Phys..

[cit31] Todorov T. N., Dundas D., McEniry E. J. (2010). Phys. Rev. B: Condens. Matter Mater. Phys..

[cit32] Bai M., Cucinotta C. S., Jiang Z., Wang H., Wang Y., Rungger I., Sanvito S., Hou S. (2016). Phys. Rev. B.

[cit33] Lu J.-T., Wang J.-S., Hedegard P., Brandbyge M. (2016). Phys. Rev. B.

[cit34] Neaton J. B., Hybertsen M. S., Louie S. G. (2006). Phys. Rev. Lett..

[cit35] Garcia-Lastra J. M., Thygesen K. S. (2011). Phys. Rev. Lett..

[cit36] Toher C., Filippetti A., Sanvito S., Burke K. (2005). Phys. Rev. Lett..

[cit37] Strange M., Rostgaard C., Häkkinen H., Thygesen K. S. (2011). Phys. Rev. B: Condens. Matter Mater. Phys..

[cit38] Pemmaraju C. D., Rungger I., Chen X., Rocha A. R., Sanvito S. (2010). Phys. Rev. B: Condens. Matter Mater. Phys..

[cit39] Souza A. M., Rungger I., Pemmaraju C. D., Schwingenschloegl U., Sanvito S. (2013). Phys. Rev. B: Condens. Matter Mater. Phys..

[cit40] Souza A. d. M., Rungger I., Pontes R. B., Rocha A. R., da Silva A. J. R., Schwingenschloegl U., Sanvito S. (2014). Nanoscale.

[cit41] Ordejón P., Artacho E., Soler J. M. (1996). Phys. Rev. B: Condens. Matter Mater. Phys..

[cit42] Soler J. M., Artacho E., Gale J. D., García A., Junquera J., Ordejón P., Sánchez-Portal D. (2002). J. Phys.: Condens. Matter.

[cit43] Perdew J. P., Burke K., Ernzerhof M. (1996). Phys. Rev. Lett..

[cit44] Dion M., Rydberg H., Schroder E., Langreth D. C., Lundqvist B. I. (2004). Phys. Rev. Lett..

[cit45] Wand J., Roman-Perez G., Soler J., Artacho E., Fernandez-Serra M. V. (2011). J. Chem. Phys..

[cit46] Troullier N., Martins J. L. (1991). Phys. Rev. B: Condens. Matter Mater. Phys..

[cit47] Michaelides A., Ranea V. A., de Andres P. L., King D. A. (2003). Phys. Rev. Lett..

[cit48] Cicero G., Calzolari A., Corni S., Catellani A. (2011). J. Phys. Chem. Lett..

[cit49] Poissier A., Ganeshan S., Fernandez-Serra M. V. (2011). Phys. Chem. Chem. Phys..

[cit50] Carrasco J., Michaelides A., Scheffler M. (2009). J. Chem. Phys..

[cit51] Huzayyin J., Chang J. H., Lian K., Dawson F. (2014). J. Phys. Chem. C.

[cit52] Note that the positive bias is defined when we have net negative charge on the surface. Therefore, this situation corresponds to the negative electric field in ref. 51

[cit53] Pedroza L. S., Poissier A., Fernández-Serra M.-V. (2015). J. Chem. Phys..

[cit54] Bader R. F. W., Atoms in Molecules: A Quantum Theory, Oxford University Press, Oxford, UK, 1994.

[cit55] Keijsers R. J. P., Voets J., Shklyarevskii O. I., van Kempen H. (1996). Phys. Rev. Lett..

[cit56] Weiss, Wagner C., Kleimann C., Rohlfing M., Tautz F. S., Temirov R. (2010). Phys. Rev. Lett..

[cit57] Feliciano G. T., Sanz-Navarro C., Coutinho-Neto M. D., Ordejón P., Scheicher R. H., Rocha A. R. (2015). Phys. Rev. Appl..

[cit58] Feliciano G. T., Sanz-Navarro C., Coutinho-Neto M. D., Ordejón P., Scheicher R. H., Rocha A. R. (2017). J. Phys. Chem. B.

