# Supplementary material for: Bias-dependent local structure of water molecules at a metallic interface
Source: Chem Sci. 2017 Oct 11;9(1):62–9. doi: 10.1039/c7sc02208e (PMC5869310; doi:10.1039/c7sc02208e)
Supplement: Supplementary file 1 [file SC-009-C7SC02208E-s001.pdf]

Electronic Supplementary Material (ESI) for Chemical Science.
This journal is © The Royal Society of Chemistry 2017

Supplementary Information: Bias-dependent local structure of water molecule at a metallic interface

Luana S. Pedroza

ICTP South American Institute for Fundamental Research,
Instituto de Física Teórica, Universidade Estadual Paulista,
São Paulo SP 01140-070, Brazil, and
Centro de Ciências Naturais e Humanas, Universidade Federal do ABC,
Santo André, São Paulo, Brazil 09210-170.

Pedro Brandimarte

Centro de Física de Materiales, 20018 Donostia-San Sebastián, Gipuzkoa Spain, and
Donostia International Physics Center,
20018 Donostia-San Sebastián, Gipuzkoa, Spain.

Alexandre Reily Rocha

Instituto de Física Teórica, Universidade Estadual Paulista,
São Paulo, São Paulo SP 01140-070, Brazil

M.-V. Fernández-Serra

Department of Physics and Astronomy, Stony Brook University,
Stony Brook, New York 11794-3800, USA, and
Institute for Advanced Computational Sciences,
Stony Brook University, Stony Brook, New York 11794-3800, USA

I. FORCES AS A FUNCTION OF BIAS

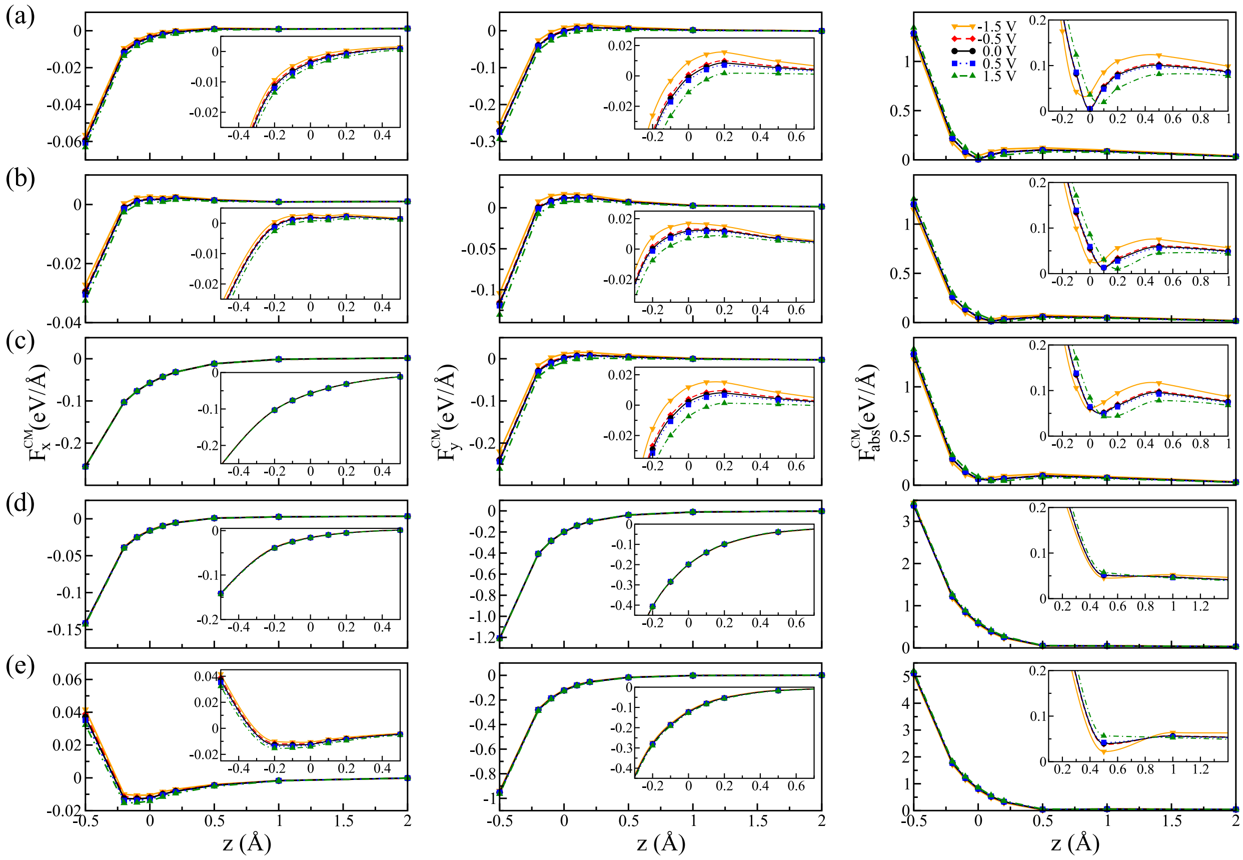

Figure S1. x- (left panel) and y-components (middle panel) of the forces on center of mass of the water molecule for the different structures considered in this work (shown in Figure 2 of the main manuscript). (right panel) Total force on the center of mass of the H_2O molecule. The z-component is presented in the main text.

II. BADER CHARGES

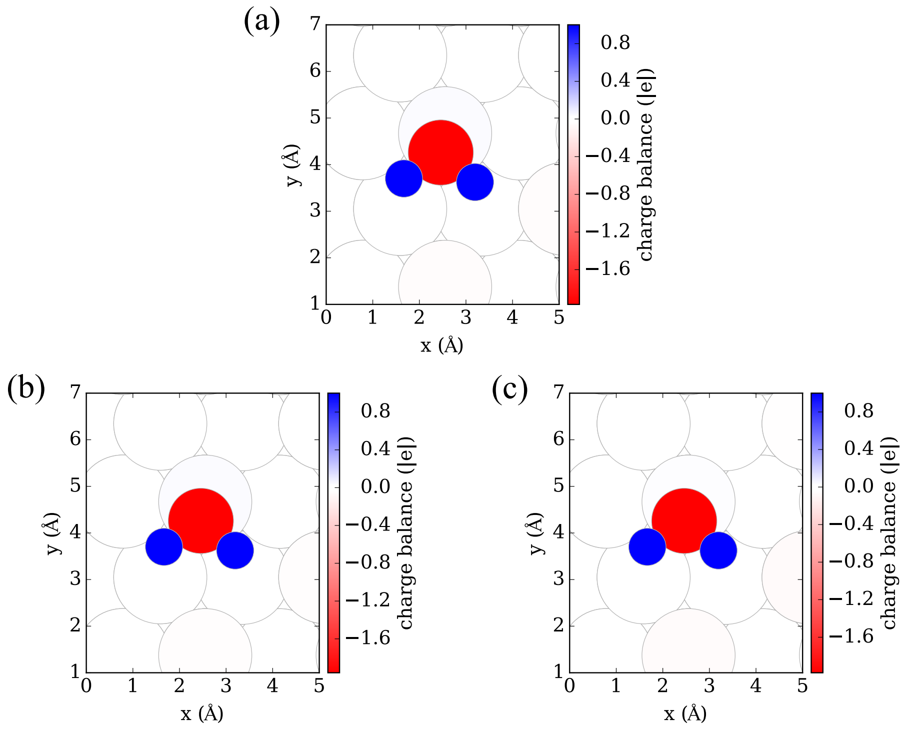

Figure S2. Bader valence charges on a water molecule adsorbed on an Au surface with different bias: (a) V = 0 V, (b) V = -1.5 V, and (c) V = 1.5 V.

III. DIPOLE MOMENT ESTIMATION

To analyze the effect of the electronic density redistribution on the water molecule we calculated the dipole moment $\boldsymbol{p}_i^{\mathbf{H_2O}}$ for a particular arrangement i. In general, the dipole moment is defined in terms of the difference in charge between the converged charge density and the corresponding atomic distributions

$$\boldsymbol{p} = \int \left(\rho_{tot}(\mathbf{r}) - \sum_{I=1}^{N_{atoms}} \rho_I(\mathbf{r}) \right) (\mathbf{r} - \mathbf{r}_0)\, d\mathbf{r} \tag{S1}$$

in such a way that

$$\int \left(\rho_{tot}(\mathbf{r}) - \sum_{I=1}^{N_{atoms}} \rho_I(\mathbf{r}) \right) \sim 0\,. \tag{S2}$$

As we are interested in the dipole moment of the water molecule alone, for each bias V, we remove the effect of an equivalent parallel plate capacitor with potential V,

$$\rho_{tot}\left(V\right) = \rho_{Au+H_2O}\left(V\right) - \left(\rho_{Au}\left(V\right) - \sum_{I=1}^{N_{Au}} \rho_I^{Au}(\mathbf{r}) \right)\,. \tag{S3}$$

All calculations are performed using $\mathbf{r}_0 = \mathbf{r}_{\mathrm{CM}}$, and, in order to guarantee charge neutrality of the charge density, the limits of integration are chosen as to satisfy equation S2.
